# Bayesian-optimized machine learning boosts actual evapotranspiration prediction in water-stressed agricultural regions of China

**DOI:** 10.1038/s41598-025-22130-y

**Published:** 2025-10-28

**Authors:** Ahmed Elbeltagi, Aman Srivastava, Xinchun Cao, Vinay Kumar Gautam, Bilel Zerouali, Muhammad Rizwan Aslam, Ali Salem, Hojjat Emami, Elsayed Ahmed Elsadek

**Affiliations:** 1 College of Agricultural Science and Engineering, Hohai University, Nanjing, 211100 China; 2 Agricultural Engineering Dept., Faculty of Agriculture, Mansoura University, Mansoura, 35516 Egypt; 3 Formerly, Centre for Technology Alternatives for Rural Areas (CTARA), Indian Institute of Technology (IIT) Bombay, Mumbai, 400076 India; 4 Department of Bioresource Engineering, Faculty of Agricultural & Environmental Sciences, McGill University, Québec, H9X 3V9 Canada; 5 School of Natural Resource Management, College of Post Graduate Studies in Agricultural Sciences, Central Agricultural University, (Imphal), Umiam, Imphal, Meghalaya 793103 India; 6 Laboratory of Architecture, Cities and Environment, Department of Hydraulic, Faculty of Civil Engineering and Architecture, University of Chlef, Hassiba Benbouali, B.P. 78C, 02180 Ouled Fares, Chlef Algeria; 7 College of Environmental and Resource Sciences, Zhejiang University, Hangzhou, 310058 China; 8 Civil Engineering Department, Faculty of Engineering, Minia University, Minia, 61111 Egypt; 9 Structural Diagnostics and Analysis Research Group, Faculty of Engineering and Information Technology, University of Pécs, Pécs, Hungary; 10 Department of Computer Engineering, Bonab University, Bonab, Iran; 11 Biosystems Engineering Department, University of Arizona, Tucson, AZ 85721 USA; 12 Agricultural and Biosystems Engineering Department, College of Agriculture, Damietta University, Damietta, 34517 Egypt

**Keywords:** Data-driven evapotranspiration, Bayesian optimization, Agricultural water management, Agricultural hydrology, Climate data modeling, Irrigation scheduling, Hydrology, Environmental impact, Climate change, Hydrology

## Abstract

The accurate estimation of actual evapotranspiration (AET) is crucial for sustainable water resource management, especially in water-scarce and agriculturally intensive regions like Beijing and Tianjin, China. Traditional methods for AET estimation, whether empirical or physically based, often face limitations due to high data requirements, limited scalability, and sensitivity to input uncertainties. This creates a critical research gap in providing reliable AET predictions under data-limited conditions. To address this, we evaluated the efficacy of integrating four advanced machine learning (ML) models: Support Vector Machine (SVM), Gaussian Process Regression (GPR), Ensemble Tree, and Neural Network, with Bayesian hyperparameter optimization for AET modeling using the high-resolution TerraClimate dataset spanning 1958–2022. Key meteorological variables, including maximum and minimum temperature (T_max_ and T_min_), solar radiation (SR), wind speed (WS), vapor pressure deficit (VPD), and precipitation (PPT), were selected through rigorous correlation and multicollinearity analyses. Model performance was assessed using the coefficient of determination (R^2^), mean squared error (MSE), root mean squared error (RMSE), and mean absolute error (MAE) on a 75:25 train-test split. Results demonstrate that the optimizable GPR model achieved the highest predictive accuracy (RMSE = 5.54, R^2^ = 0.98 on test data), outperforming other ML approaches and traditional empirical models. PPT, T_min_, and T_max_ emerged as the most influential predictors for AET. Our findings reveal that ML models, particularly when optimized via Bayesian techniques, yield a robust, scalable, and data-efficient alternative for AET estimation in regions with limited meteorological records. This study establishes a new benchmark for AET modeling, with significant implications for irrigation scheduling, drought monitoring, and integrated water management in the North China Plain and comparable agro-ecological regions.

## Introduction

Water is a finite and essential resource that plays a pivotal role in sustaining ecosystems and supporting human activities. Agricultural water management becomes serious given the continued rise of global water demand and climate change. At the core of this management is the intricate process of evapotranspiration (ET), a critical component of the water cycle^1,2^ involving the combined water loss from soil and plant surfaces^3–5^, which is profoundly affected by climate change. Climate change modifies global weather patterns, leading to extreme shifts in temperatures and precipitation^6^. These cause significant disruptions to crop production^7^, especially in arid and semi-arid regions, where drought conditions accelerate water scarcity^6,8^. Thus, understanding and quantifying ET become paramount for enhancing water resources management and supporting decision-making^9,10^ under such conditions.

Determining crop ET involves adopting a nuanced approach that integrates direct and indirect methods. On one hand, direct techniques, such as employing the pan evaporation method and lysimeter measurements^11^, conducting experiments in field plots^12^, implementing moisture reduction control^13^, and using flux networks (called FLUXNET)^14^, deliver precise, on-site data. Nevertheless, there are scaling issues and measurement errors associated with these methods^15^. On the other hand, indirect methods, encompassing the aerodynamic approach^16^, energy balance calculations^17^, and their synergistic application, generate estimates based on atmospheric and energy-related parameters such as temperature-based models (Hargreaves, Thornthwaite, and Blaney-Criddle), radiation-based models (Christiansen, and Doorenbos-Pruitt), and combination methods (FAO-56 Penman–Monteith and Hargreaves-Samani)^18–21^. However, practical considerations often influence the choice between direct and indirect methods. Direct methods, while offering detailed insights, tend to be both costly and time-consuming. In contrast, indirect methods present a more resource-efficient alternative for estimating evapotranspiration^22^, making them attractive for large-scale and long-term studies^23^. However, indirect methods require proper measurements of multiple meteorological variables^24^.

A paradigm shift has occurred in recent years with the rise of artificial intelligence (AI) and machine learning (ML) algorithms^25,26,76^. This revolution has significantly boosted the accuracy of evapotranspiration estimation compared to classical models, including empirical models. The dynamic capabilities of AI and ML algorithms allow for more intricate pattern recognition and data analysis, enabling a more precise understanding of the complex processes governing evapotranspiration^27^. The ML algorithms have significantly transformed the prediction of climatic series, particularly in scenarios where data is limited, as exemplified in the case of evapotranspiration^28^. Traditional approaches faced challenges in making accurate predictions under constrained data availability, but machine learning has introduced innovative methodologies to address these limitations^29^. ML algorithms excel in learning patterns and relationships from existing data, enabling them to make predictions even with limited datasets, where historical and comprehensive datasets may not always be readily available.

Recent advances in AI-ML have significantly improved the estimation of reference evapotranspiration (ET_o_) and actual evapotranspiration (AET) across diverse climatic and data availability scenarios. For instance, Rai et al.^30^ evaluated support vector regression (SVM), M5P model tree, and random forest (RF) against empirical models for monthly ET_o_ estimation in Uttarakhand and Uttar Pradesh, India, finding that the SVM-1 model with a C-1 input combination outperformed other approaches. Agrawal et al.^31^ tested five machine learning models, including Decision Tree, RF, AdaBoost, GBM, and XGBoost, and reported that ensemble models, particularly XGBoost, achieved high accuracy with a weighted standard error below 0.17 mm day^−1^. Niu et al.^32^ compared an artificial neural network using the improved Levenberg–Marquardt (LM) algorithm with a genetic algorithm-optimized backpropagation network (GA-BP), reporting superior performance for the LM model, especially when wind speed was included. Chia et al.^33^ addressed the data-intensive nature of ML models by introducing ensemble approaches, such as non-linear neural ensemble-based inter-model ensemble (NNE-E) and Bayesian modeling (BMA-E), which enabled robust daily ET_o_ estimation even with reduced meteorological inputs. Recent developments in the Bayesian algorithm have further enhanced feature selection efficiency due to increased computational power, algorithmic optimizations, and integration with parallelization and popular ML frameworks^34,35^. Bayesian’s versatility and accuracy have been demonstrated across large datasets and diverse domains, including bioinformatics, image analysis, and natural language processing. Collectively, these studies highlight the growing capability of machine learning and data-driven feature selection to improve evapotranspiration modeling, especially under data-limited conditions and complex environmental settings.

Despite the essential role of ET estimation in sustainable water management and agricultural planning, current research still shows gaps in the spatial and temporal resolution of ET products. Additionally, there is a lack of comprehensive comparative evaluations of advanced ML models under diverse environmental conditions, such as those found in the North China Plain. Most existing studies have either focused on potential or reference ET, relying on empirical or physically-based models that require extensive meteorological inputs, which limit their applicability in data-scarce regions. Furthermore, the explainability and interpretability of ML models for ET prediction remain insufficiently explored, constraining their adoption in operational water management. This study addresses these knowledge gaps by systematically evaluating and optimizing multiple state-of-the-art ML models, including an optimizable support vector machine, Gaussian process regression, ensemble tree, and neural network-for actual evapotranspiration (AET) prediction in the highly water-stressed and agriculturally vital cities of Tianjin and Beijing. By integrating the high-resolution TerraClimate dataset (1958–2022), we hypothesize that Bayesian-optimized ML models can significantly boost AET prediction accuracy and robustness compared to traditional approaches, even with limited meteorological data. The specific objectives are to (1) identify the most influential climatic variables for AET prediction in the study region, (2) benchmark the predictive performance of different ML models using rigorous statistical metrics, and (3) provide an understanding of the practical deployment of data-driven AET modeling for integrated urban-agricultural water management. The novelty of this research lies in its comprehensive comparison of Bayesian-optimized ML models for AET prediction at a regional scale, its focus on AET rather than potential or reference ET, and its demonstration of scalable, explainable approaches suitable for data-constrained environments.

## Materials and methods

### Study area

**Fig. 1:**
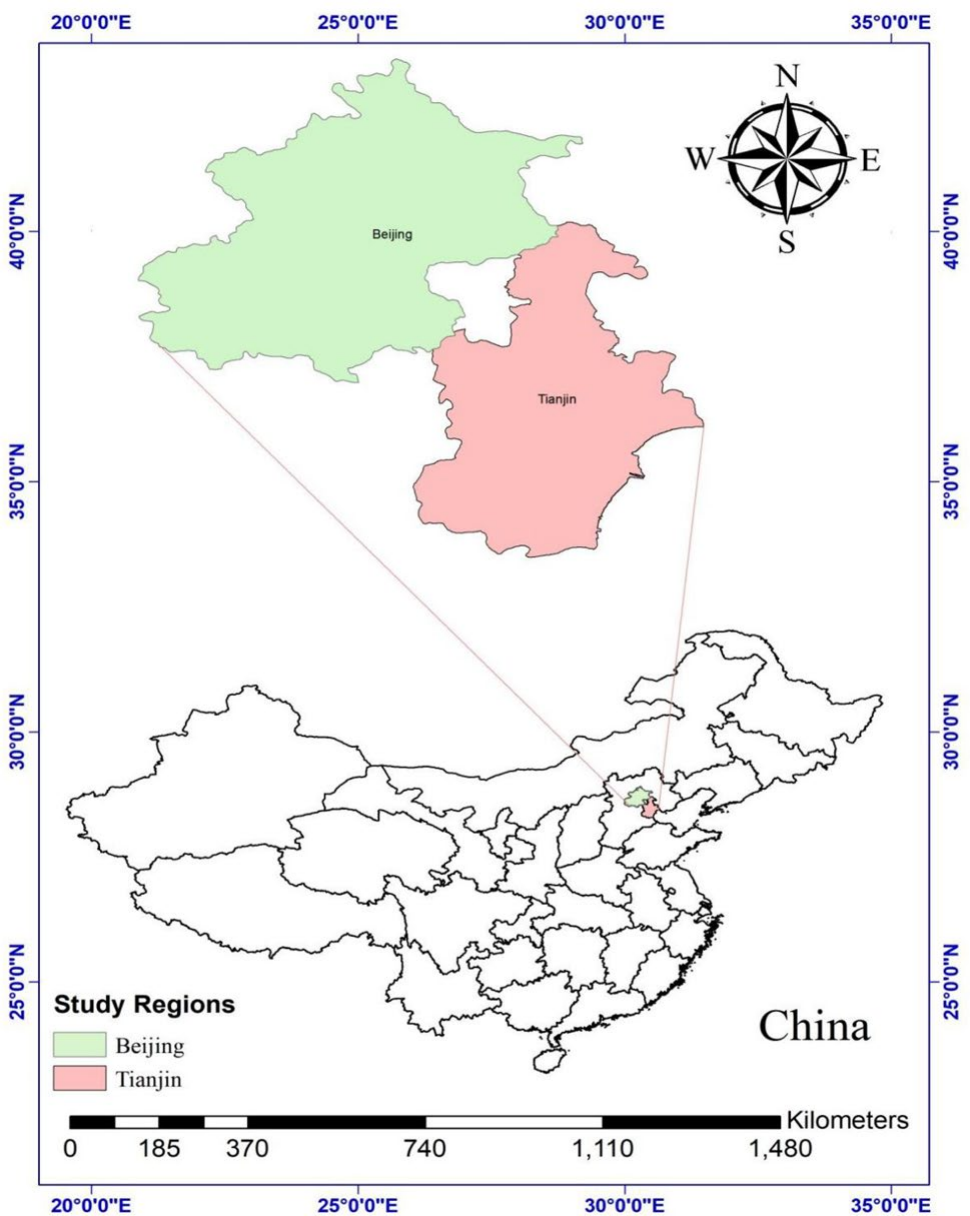
Map of the study area with respect to mainland China.

The study area, Beijing and Tianjin cities, is located in the North China Plain (NCP), the largest agricultural production region in China, with an average annual temperature reaching 15 °C and precipitation ranging between 500 and 1000 mm^36^ (Fig. 1). Both cities are among the most significant cities in the country due to their political and economic influence^37^ and their pivotal roles in water resources management and agriculture. Despite advancements in desalination, wastewater treatment, and industrial water recycling, the per capita water resources are still markedly below the national average in both cities. Moreover, the spatial and temporal distribution of these resources is uneven^38^. Therefore, effective water management in Beijing and Tianjin is crucial for ensuring food and water security across the entire region. In this context, investments in green infrastructure, urban–rural coordination, and ecosystem restoration are vital for integrating climate resilience into water and agricultural policies. The accurate estimation of evapotranspiration (ET) facilitates improved water allocation among agriculture, industry, and urban use. Given that over-extraction of groundwater constitutes a significant issue, ET data is instrumental in evaluating actual water consumption and promoting sustainable groundwater management. Moreover, ET estimation supports early drought detection, enabling authorities to implement preventive measures.

### Dataset sources

**Fig. 2:**
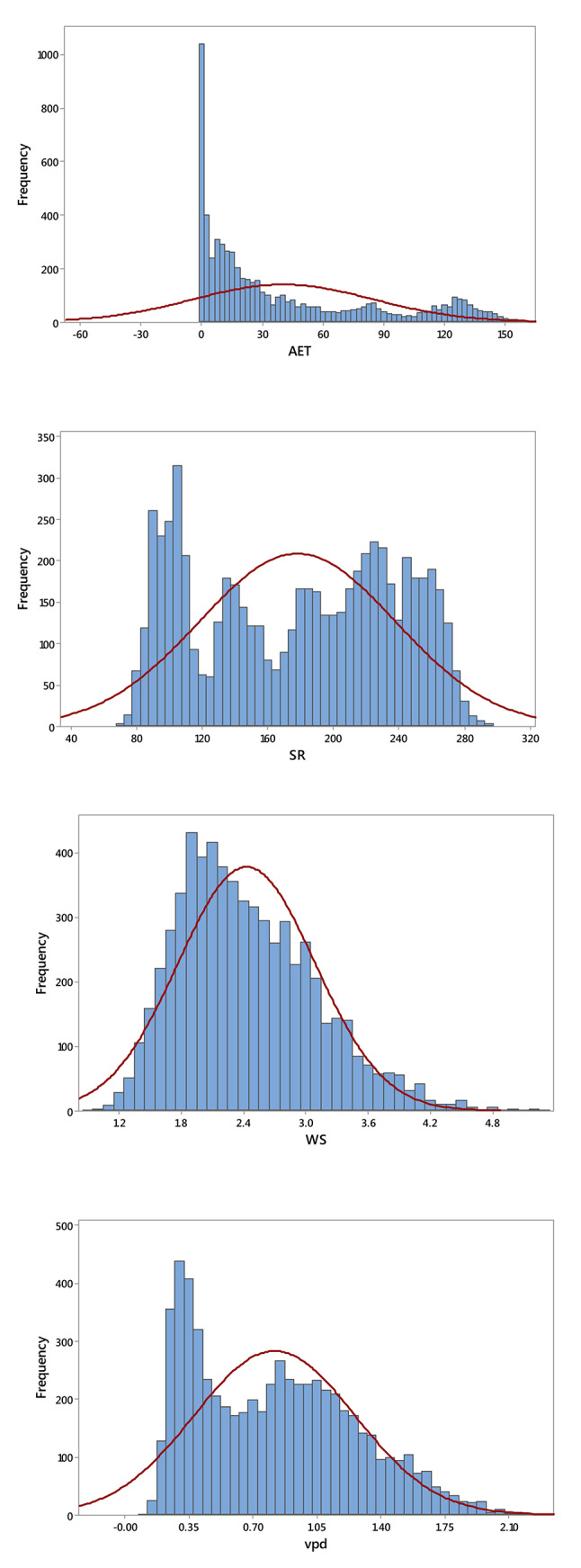

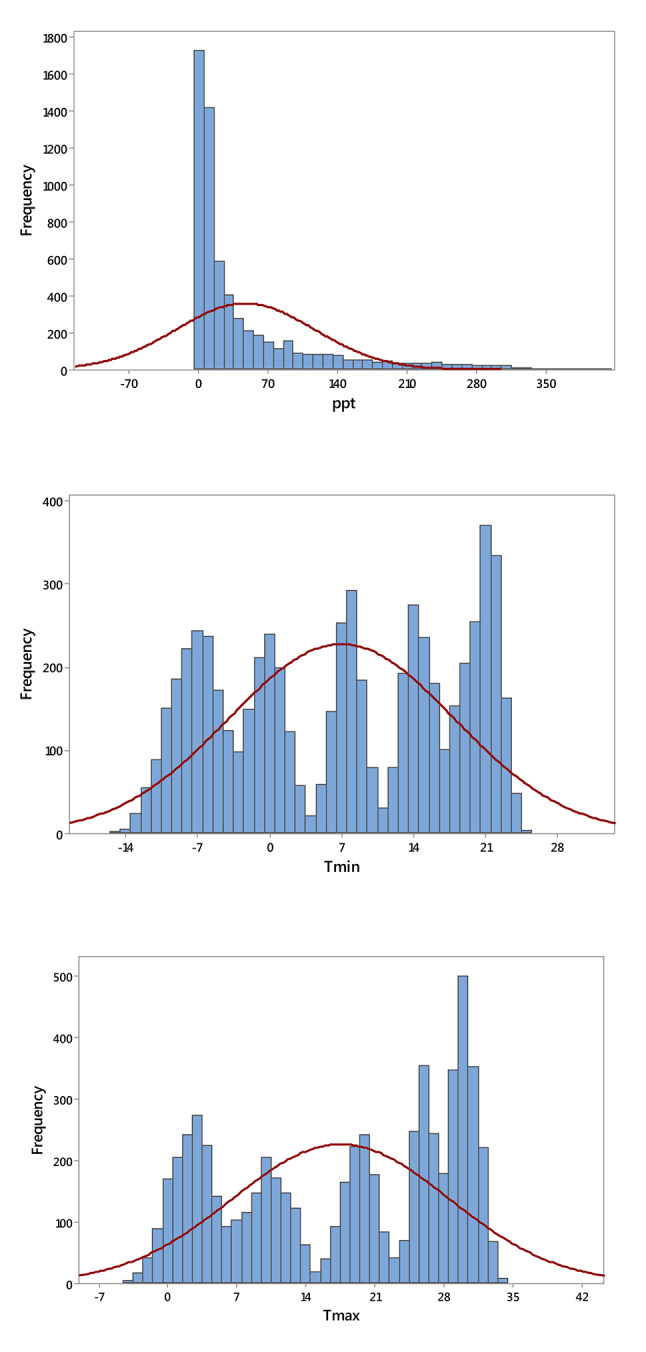
Probability distribution histograms of key climate variables and actual evapotranspiration (AET) in the study area. T_max,_ T_min_, and PPT refer to maximum, minimum temperature in °C, and precipitation in mm. VPD is vapor pressure deficit in kPa, WS is wind speed in m·s^−1^, SR is daily total shortwave radiation in MJ· m^−2^ ·day^−1^, and AET is actual evapotranspiration in mm.

The TerraClimate dataset provides monthly actual evapotranspiration (AET) data for all global land surfaces, covering the period from 1958 to 2022^39,40^. For the regions studied in China, the dataset includes key climatological parameters: maximum temperature (T_max_, °C), minimum temperature (T_min_, °C), solar radiation (SR, MJ m^−2^ ·day^−1^), wind speed (WS, m·s^−1^), vapor pressure deficit (VPD, kPa), and precipitation (PPT, mm). These variables are essential for analyzing long-term variations in AET. With a monthly temporal resolution and a spatial resolution of approximately 4 km (1/24°), TerraClimate is widely used in hydrology, water resource management, and ecological research due to its detailed spatial and temporal coverage. The dataset is generated using a climatically aided interpolation method, which combines coarser, time-varying data from CRU Ts4.0 and the Japanese 55-year Reanalysis (JRA55) with high-resolution climatological normals from WorldClim^41,42^. This approach interpolates temporal anomalies from CRU and JRA55 onto the high-resolution WorldClim baseline, resulting in a dataset that offers both fine spatial detail and a long temporal record. Most terrestrial areas on Earth receive their time-series data for temperature, precipitation, and vapor pressure from the CRU Ts4.0 dataset. However, in regions where CRU lacks climate station input, such as Antarctica, certain parts of Africa and South America, and some isolated islands, the JRA55 reanalysis dataset is used instead. TerraClimate also documents the number of climate stations (ranging from 0 to 8) contributing to the CRU Ts4.0 data for temperature, vapor pressure, and precipitation. For solar radiation and wind speed, TerraClimate relies solely on JRA55 data. In addition to climatological variables, TerraClimate generates monthly surface water balance estimates using advanced modeling techniques that incorporate reference evapotranspiration, precipitation, temperature, and plant-available soil water capacity. Validation of the TerraClimate dataset has been robust, drawing on extensive station-based observations from multiple networks, including SNOTEL, RAWS, and the Global Historical Climatology Network (GHCN). While validation metrics, particularly error measures, were marginally better for the original CRU Ts4.0 data, TerraClimate exhibited improved geographic realism. Moreover, a strong correlation was observed between TerraClimate’s annual in-situ reference evapotranspiration estimates and those recorded at FLUXNET stations. Table 1 presents a descriptive statistic of climatic variables obtained from the meteorological station, showing variability in different parameters. Additionally, Fig. 2 depicts the frequency distribution of different climatic variables in the dataset used in the current study.

**Table 1:** Descriptive statistics of meteorological data.

Variable	Mean	SE	SD	Min	Q1	Median	Q3	Max
T_max,_ °C	17.57	0.14	11.01	− 4.41	7.08	19.54	28.13	34.08
T_min,_ °C	7.00	0.14	10.95	− 15.05	− 2.96	7.67	17.25	24.90
PPT, mm	46.67	0.88	69.50	0.00	4.20	14.60	58.08	405.50
VPD, kPa	0.82	0.01	0.44	0.11	0.41	0.80	1.13	2.28
WS, m·s^−1^	2.43	0.01	0.66	0.92	1.93	2.33	2.85	5.29
SR, MJ· m^−2^ ·day^−1^	178.21	0.76	59.83	71.10	118.20	184.80	229.60	295.50
AET, mm	40.18	0.56	44.48	0.00	5.10	20.50	66.80	160.20

SE and SD are the standard error and deviation, respectively. In a box plot, Q1 (the first quartile) and Q3 (the third quartile) define the box itself, marking the boundaries of the interquartile range (IQR). Max is the maximum value. T_max,_ T_min_, and PPT refer to maximum, minimum temperature, and precipitation. VPD is vapor pressure deficit, WS is wind speed, SR is daily total shortwave radiation, and AET is actual evapotranspiration.

### Machine learning models

#### Optimizable SVM

An optimizable Support Vector Machine (SVM) refers to a machine learning model that automatically fine-tunes its hyperparameters during training to achieve optimal performance^43,44^. This approach combines the robust classification capabilities of traditional SVMs with automated hyperparameter optimization techniques, resulting in a model that is more accurate, adaptable, and efficient, especially when applied to complex or high-dimensional datasets. Support Vector Machines are a type of supervised learning algorithm used for classification and regression tasks. SVMs work by identifying the optimal hyperplane that separates classes of data in a feature space. The goal is to maximize the margin between different classes, ensuring robust classification. An optimizable SVM uses automated optimization algorithms to search for the best combination of these hyperparameters. This is particularly valuable because the choice of hyperparameters can significantly impact model performance, and manual tuning is time-consuming and may not yield the best results.

#### Optimizable Gaussian Process Regression (GPR)

Gaussian Process Regression (GPR) is a non-parametric, probabilistic model used for regression tasks. It provides a flexible framework for making predictions on continuous data. Unlike traditional regression models, GPR doesn’t assume a specific parametric form for the underlying function and instead uses a Gaussian process to model the data^45,46^. An optimizable Gaussian Process Regression model refers to a GPR where the hyperparameters of the kernel function are automatically optimized during the training process. In standard GPR, the kernel hyperparameters are usually chosen manually based on domain knowledge or empirical trials. However, optimizing these hyperparameters is essential for improving the model’s predictive accuracy and generalization ability. The optimization of GPR’s hyperparameters can be approached in various ways, such as Bayesian optimization used in this work. This is an advanced optimization technique that builds a probabilistic model to guide the search for optimal hyperparameters. Bayesian optimization is particularly useful when the evaluation of the objective function (in this case, the marginal likelihood) is expensive or time-consuming. The process of optimizing a Gaussian Process Regression model typically involves the following steps: 1) Initialization: Select a kernel function and initial values for the kernel’s hyperparameters (e.g., length scale, variance). These initial values can be chosen based on prior knowledge or set randomly; 2) Train the model: Using the training data, calculate the marginal likelihood of the model. This likelihood expresses how likely the observed data is, given the kernel and hyperparameters; 3) Hyperparameter optimization: Use optimization techniques such as MLE, Bayesian optimization, or gradient-based optimization to adjust the kernel hyperparameters. The goal is to maximize the marginal likelihood and find the best hyperparameters; 4) Model evaluation: After optimizing the hyperparameters, the model is evaluated using a test dataset to assess its predictive performance. This may involve computing metrics such as mean squared error (MSE), root mean squared error (RMSE), or coefficient of determination (R^2^); 5) Iterate: In some cases, the optimization process may be iterated multiple times, refining the hyperparameters based on the validation performance until optimal results are achieved.

#### Optimizable Ensemble Tree

Ensemble learning is a machine learning paradigm that combines multiple individual models (or base learners) to create a stronger, more accurate prediction. The main idea behind ensemble learning is that by combining several weak models, the ensemble can make better predictions than any individual model could on its own^47–49^. The Ensemble Tree is a type of ensemble model that uses decision trees as its base learners. Decision trees are simple, interpretable models that recursively split the feature space into regions based on feature thresholds. However, individual decision trees tend to overfit the data and perform poorly on unseen data. To mitigate this, ensemble tree methods combine multiple decision trees to form stronger models. The optimizable Ensemble Tree refers to an ensemble learning model where the hyperparameters of the decision trees and the ensemble method itself are automatically optimized. These hyperparameters can significantly impact the performance of the model, whereas optimizing them allows the ensemble to learn better patterns, increase generalization, and reduce overfitting. The optimization of GPR’s hyperparameters can be approached in various ways, such as Bayesian optimization used in this study. This is an advanced optimization technique that builds a probabilistic model to guide the search for optimal hyperparameters. Bayesian optimization is particularly useful when the evaluation of the objective function (in this case, the marginal likelihood) is expensive or time-consuming.

#### Optimizable neural network

A neural network is a computational model inspired by the way biological neural networks in the brain process information. In machine learning, neural networks are used for a wide variety of tasks, such as classification, regression, and pattern recognition. The basic structure of a neural network consists of layers of interconnected nodes/neurons, where each node in a layer is connected to nodes in the subsequent layer by weighted connections^50,51^. An optimizable neural network refers to a neural network whose hyperparameters (such as learning rate, number of layers, number of neurons per layer, activation functions, etc.) can be automatically tuned to improve the model’s performance. Optimizing the hyperparameters is a critical part of training deep learning models, as these choices significantly affect the model’s ability to generalize and achieve high accuracy. Optimizing the hyperparameters of a neural network can be challenging due to the high-dimensional nature of the search space. Bayesian optimization, used as an optimization technique in this study, is a probabilistic model-based method that uses previous evaluations to guide the search for the best hyperparameters. It builds a surrogate model (often a Gaussian Process) to predict the performance of different hyperparameter combinations. Here, “optimizable” implies that these models go through a methodical tuning procedure to determine the best hyperparameter setups. The algorithms are optimized for maximum performance in forecasting AET by utilizing optimization strategies specific to each type of model. Hyperparameters for tuning and optimization for different models are summarized in Table 2. The datasets were partitioned into training and testing sets using a 75:25 split, where 75% of the data was used for training the model and the remaining 25% was reserved for testing its performance. This division ensures that the model learns from a substantial portion of the data while still being evaluated on unseen data to assess its generalization ability.

**Table 2:** Hyperparameter configurations and optimization strategies employed for machine learning models in actual evapotranspiration (AET) estimation.

Model type	Hyperparameters
Optimizable SVM	None; Optimizer: Bayesian optimization; Acquisition function: Expected improvement per second plus; Iterations: 30; Training time limit: false
Optimizable Gaussian Process Regression	Signal standard deviation: 31.4281; Optimize numeric parameters: Yes; Optimizer: Bayesian optimization; Acquisition function: Expected improvement per second plus; Iterations: 30; Training time limit: false
Optimizable Ensemble Tree	Optimizer: Bayesian optimization; Acquisition function: Expected improvement per second plus; Iterations: 30; Training time limit: false
Optimizable neural network	Iteration limit: 1000; Optimizer: Bayesian optimization; Acquisition function: Expected improvement per second plus; Iterations: 30; Training time limit: false

SVM refers to support vector machine.

#### Hyperparameter optimization and model parameter settings

To ensure robust, accurate, and generalizable predictions of AET, we implemented a comprehensive hyperparameter optimization protocol leveraging Bayesian optimization for all machine learning models assessed in this study. Bayesian optimization was selected due to its proven efficiency and effectiveness when exploring complex, multidimensional hyperparameter spaces, outperforming traditional tuning methods such as exhaustive grid search or random search. It works by building a probabilistic surrogate model of the objective function—here, the prediction error—and iteratively selecting hyperparameter candidates that balance exploration of unexplored regions with exploitation of promising areas in the parameter space.

Specifically, we employed the “Expected Improvement per Second plus” acquisition function during Bayesian optimization. This choice enables an optimal trade-off between the probability of improving upon current best results and the computational time required per evaluation, allowing efficient convergence toward near-optimal hyperparameter configurations within limited iterations. For each model, the optimization ran for 30 iterations, an empirically derived setting striking a balance between thorough hyperparameter space exploration and computational cost, making it suitable for practical applications in hydrological forecasting.

Hyperparameter tuning for individual models

*SVM:* The model used a Radial Basis Function (RBF) kernel, which is well-suited for capturing the nonlinear relationships typical in AET modeling. Key hyperparameters, including the penalty parameter (C), which governs the trade-off between margin maximization and error minimization, and the kernel scale (defining the influence radius of data points), were optimized within the Bayesian framework. According to Table 2, while no fixed hyperparameters were preset, Bayesian optimization guided these selections for each iteration, without a preset training time limit.

*GPR:* The GPR model utilized a squared exponential kernel, appropriate for modeling the smooth, continuous variations expected in climatic drivers of evapotranspiration. The signal standard deviation was empirically optimized to approximately 31.43. Bayesian optimization was applied to numerically optimize kernel parameters, including length scales and noise levels, essential for balancing fit accuracy and uncertainty quantification accuracy. Optimization ran for 30 iterations under the same acquisition function with no time limits, consistent with other models.

*Ensemble Tree:* This gradient boosting tree model had hyperparameters such as the number of learners (trees), maximum number of splits per tree (depth control), and learning rate optimized. The Bayesian optimization similarly ran for 30 iterations using the same acquisition function and no training time restrictions, effectively balancing bias and variance to prevent overfitting in the presence of correlated inputs.

*Neural network:* The neural network model’s hyperparameters, which included the number of hidden layers and neurons, learning rate, regularization coefficients, and training epochs, were optimized with an iteration limit of 1000 epochs during training. Bayesian optimization controlled the search over these parameters across 30 iterations to enhance model convergence and avoid overfitting, while maintaining compatibility with dataset size and complexity.

*Train-test split*: For model training and validation, a randomized train-test split was applied where approximately 75% of the dataset was reserved for training and hyperparameter tuning, while the remaining 25% was kept aside exclusively for independent testing to evaluate model generalizability. This split ratio was chosen to provide ample data for model fitting while maintaining an unseen test subset for unbiased accuracy assessment. To mitigate potential bias from a single random partition, the procedure was repeated across mutible randomized splits, and model performance was reported as the mean and standard deviation across these runs. This repeated randomization helps reduce the influence of temporal autocorrelation and provides a more robust estimate of predictive performance. Collectively, this rigorously designed approach to hyperparameter optimization, model parameter selection, and data partitioning ensures that the machine learning models developed here are not only computationally efficient but also well-calibrated to capture the complex nonlinear dependencies between climatic factors and AET in the water-stressed, heterogeneous agricultural environments of Beijing and Tianjin. Hyperparameter tuning was performed in MATLAB R2024b (Statistics and Machine Learning Toolbox) using Bayesian optimization with the expected-improvement-plus acquisition function. For each model, we allowed a maximum of 30 objective evaluations (MaxObjectiveEvaluations = 30). Each candidate setting was assessed using fivefold cross-validation within the training set (Kfold = 5) to mitigate overfitting and select the model that minimized cross-validated loss. Continuous positive parameters were searched on a log scale; categorical choices were explored uniformly. 

### Performance metrics

Evaluating the prediction error rate within a regression model provides essential insights into its overall efficacy. A regression model is regarded as effective when the discrepancies between actual and predicted values are minimal and consistent across the training, validation, and testing datasets. In this analysis, four statistical metrics are utilized to assess the model’s precision and dependability, namely, the coefficient of determination (R^2^), mean squared error (MSE), root mean squared error (RMSE), and mean absolute error (MAE).

1 $$R^{2} = \left[ {\frac{{\sum\nolimits_{i = 1}^{n} {\left( {Y_{act} - Y_{act}^{ - } } \right)\left( {Y_{p} - Y_{p}^{ - } } \right)} }}{{\sqrt {\sum\nolimits_{i = 1}^{n} {\left( {Y_{act} - Y_{act}^{ - } } \right)^{2} \sqrt {\sum\nolimits_{i = 1}^{n} {\left( {Y_{p} - Y_{p}^{ - } } \right)^{2} } } } } }}} \right]^{2}$$

2 $${ }MSE = \frac{1}{n}\mathop \sum \limits_{i = 1}^{n} \left( {Y_{ act} - { }Y_{ p} } \right)^{2}$$

3 $${ }RMSE = \sqrt {\frac{1}{n}\mathop \sum \limits_{i = 1}^{n} \left( {Y_{ act} - { }Y_{ p} } \right)^{2} { }}$$

4 $$MAE = \frac{1}{n}\mathop \sum \limits_{i = 1}^{n} \left| {Y_{p} - Y_{act} } \right|$$

where ${Y}_{ act}$ is the observed value, ${Y}_{ p}$ is the predicted (modeled) value, and *n* is the number of values. ${{Y}_{act}}^{-}$ and ${{Y}_{p}}^{-}$ are the means of observed and predicted values, respectively.

## Results

### Cross-correlation analysis for input selection in AET modeling

Identifying the most influential climatic variables is a foundational step in developing robust ML models for AET prediction, particularly under conditions of limited data availability. In this study, we employed cross-correlation analysis to systematically assess the linear associations between AET and key meteorological variables-maximum temperature (T_max_), minimum temperature (T_min_), precipitation (PPT), vapor pressure deficit (VPD), wind speed (WS), and shortwave radiation (SR)-using the XLSTAT 2022 platform. The correlation matrix (Table 3) reveals that AET exhibits the strongest positive correlations with precipitation (r = 0.89), minimum temperature (r = 0.82), and maximum temperature (r = 0.76). Moderate positive associations are observed with shortwave radiation (r = 0.58) and vapor pressure deficit (r = 0.47), while wind speed is negatively correlated with AET (r =  − 0.34). These findings highlight the dominant roles of temperature and precipitation in driving evapotranspiration dynamics across diverse agro-climatic settings. The strong correlations of AET with PPT, T_min_, and T_max_ suggest that these variables are the most informative predictors for subsequent ML modeling in this region.

**Table 3:**
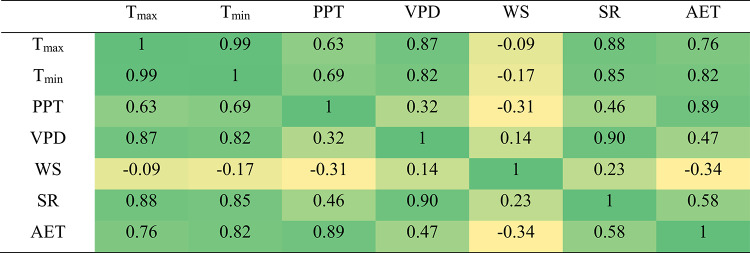
Pearson correlation matrix among climatic predictor variables and actual evapotranspiration (AET) in the study area.

T_max,_ T_min_, and PPT refer to maximum, minimum temperature, and precipitation. VPD is vapor pressure deficit, WS is wind speed, SR is daily total shortwave radiation, and AET is actual evapotranspiration.

In addition to statistical correlations, it is critical to elucidate the underlying physical processes by which these climatic factors govern actual evapotranspiration. Temperature, expressed through both maximum (T_max_) and minimum (T_min_) values, fundamentally drives AET by regulating the energy available for water vaporization from soil and vegetation surfaces^52^. Higher temperatures increase vapor pressure deficits and promote greater latent heat flux, intensifying evapotranspiration rates^53^. Precipitation (PPT) directly influences soil moisture availability, which constrains the evaporative demand limited by water supply; thus, wetter conditions typically elevate AET up to the threshold imposed by atmospheric demand^54^. Vapor pressure deficit (VPD) reflects atmospheric dryness and acts as a key controlling variable for plant transpiration, with higher VPD values enhancing transpiration fluxes, especially under adequate soil moisture conditions^55^. Meanwhile, shortwave radiation (SR) provides the primary energy input powering the evapotranspiration process through radiation absorption and redistribution within the soil–plant–atmosphere continuum^56^. Wind speed (WS) modulates the aerodynamic resistance around leaf surfaces and soil pores, influencing the efficiency of vapor removal and thus affecting the transpiration and evaporation rates^52^. Collectively, these intertwined mechanisms highlight how the principal climate variables interact physically to shape AET dynamics, justifying their dominant predictive power identified in the present correlation and machine learning analyses.

### Feature selection and multicollinearity diagnostics in AET model development

**Fig. 3:**
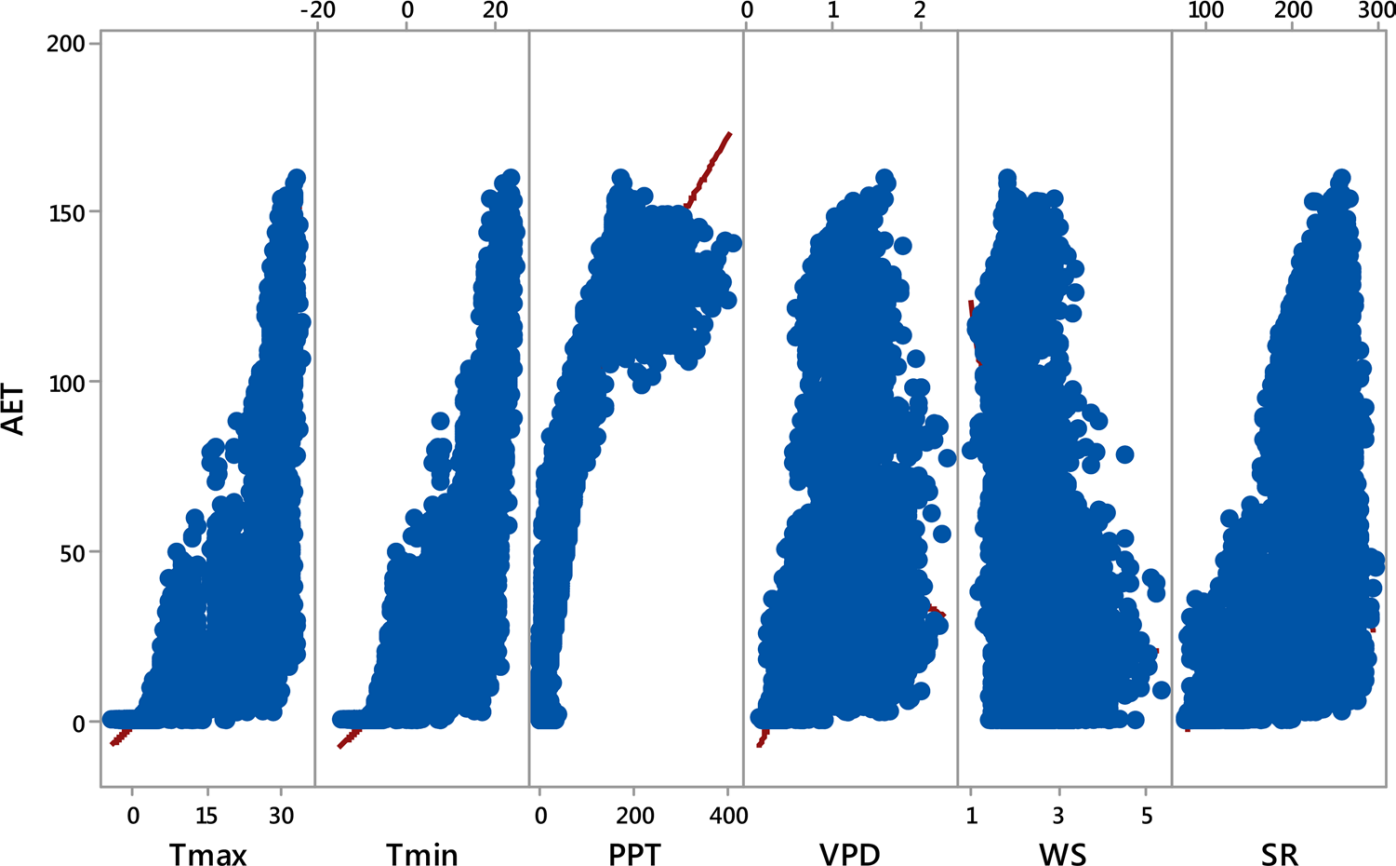
Pairwise matrix plot (scatterplot matrix) of climatic variables and actual evapotranspiration (AET) for the study area. T_max,_ T_min_, and PPT refer to maximum, minimum temperature in °C, and precipitation in mm. VPD is vapor pressure deficit in kPa, WS is wind speed in m·s^−1^, SR is daily total shortwave radiation in MJ· m^−2^ ·day^−1^, and AET is actual evapotranspiration in mm.

Multicollinearity analysis revealed critical limitations in interpreting individual climatic predictors’ contributions to AET estimation (Table 4). T_max_ and T_min_ exhibited severe multicollinearity, with variance inflation factor (VIF) values exceeding 90 and tolerance values approaching 0.01, far beyond the conventional threshold of VIF > 10 for problematic collinearity. VPD and SR showed moderate collinearity (VIF: 9.32–10.53), while PPT and WS demonstrated acceptable independence (VIF < 3.25). The matrix plot (Fig. 3) corroborated these findings, showing near-perfect linear alignment between T_max_ and T_min_ (r = 0.99), and strong associations between SR and VPD (r = 0.90). Despite these inter-variable correlations, the three most influential AET predictors identified in Sect. “Cross-Correlation Analysis for Input Selection in AET Modeling”, PPT (r = 0.89), T_min_ (r = 0.82), and T_max_ (r = 0.76), retained statistically significant relationships with the target variable. This paradoxical result highlights the need for caution when interpreting feature importance in collinear systems, even when using ML models inherently robust to multicollinearity. These diagnostics informed subsequent model development by quantifying the trade-off between predictive power and interpretability. While the extreme collinearity between temperature variables (T_max_/T_min_) could theoretically distort linear regression coefficients, the Bayesian-optimized ML models demonstrated resilience to these effects, as evidenced by their consistent performance metrics across training and testing phases (Sect. “Comparative Performance Evaluation of Bayesian-Optimized ML Models”).

**Table 4:** Multicollinearity statistics analysis.

	T_max_	T_min_	PPT	VPD	WS	SR
Tolerance	0.01	0.01	0.31	0.11	0.49	0.10
VIF	101.08	90.42	3.25	9.32	2.04	10.53

VIF is the variance inflation factor, T_max,_ T_min_, and PPT refer to maximum, minimum temperature, and precipitation. VPD is vapor pressure deficit, WS is wind speed, and SR is daily total shortwave radiation.

### Comparative performance evaluation of Bayesian-optimized ML models

The Bayesian-optimized GPR model demonstrated superior performance in predicting AET, achieving the lowest RMSE (5.54 mm), MSE (30.67 mm^2^), and MAE (2.72 mm) during testing, alongside an R^2^ of 0.98 (Table 5). This robust performance persisted across both training and testing phases, with GPR outperforming SVM, Ensemble Tree, and Neural Network models by margins of 7–37% in RMSE. The SVM and Ensemble Tree models exhibited comparable accuracy (testing RMSE: 6.00–6.27 mm), while the Neural Network lagged significantly (RMSE: 8.85 mm), likely due to overfitting or inadequate hyperparameter tuning despite Bayesian optimization (Fig. 6). Notably, all models maintained consistent R^2^ values ($\ge$ 0.96), suggesting strong explanatory power for AET variability. However, the Neural Network’s higher error metrics (MAE: 6.06 mm) reveal limitations in capturing nonlinear relationships between climatic drivers and AET in this dataset. The GPR’s success aligns with its inherent capacity to model uncertainty and complex interactions through kernel-based probabilistic frameworks, making it particularly suited for hydrological systems with limited data.

**Table 5:** Statistical performance metrics for Bayesian-optimized machine learning (ML) models in actual evapotranspiration (AET) estimation during the (A) training and (B) testing phases.

Model type	RMSE	MSE	R^2^	MAE
*A. Training stage*				
Optimizable SVM	6.52	42.49	0.98	3.21
**Optimizable Gaussian Process Regression**	**5.91**	**34.88**	**0.98**	**3.02**
Optimizable Ensemble Tree	6.75	45.59	0.98	3.67
Optimizable neural network	9.16	83.95	0.96	6.28
*B. Testing stage*				
Optimizable SVM	6.00	36.01	0.98	3.01
**Optimizable Gaussian Process Regression**	**5.54**	**30.67**	**0.98**	**2.72**
Optimizable Ensemble Tree	6.27	39.31	0.98	3.38
Optimizable neural network	8.85	78.31	0.96	6.06

The best ML model during the training and testing period is highlighted in bold. SVM refers to Support Vector Machine. RMSE is the Root Mean Square Error (mm), MSE is the Mean Squared Error (mm^2^), R^2^ is the coefficient of determination, and MAE is the Mean Absolute Error (mm).

**Fig. 4:**
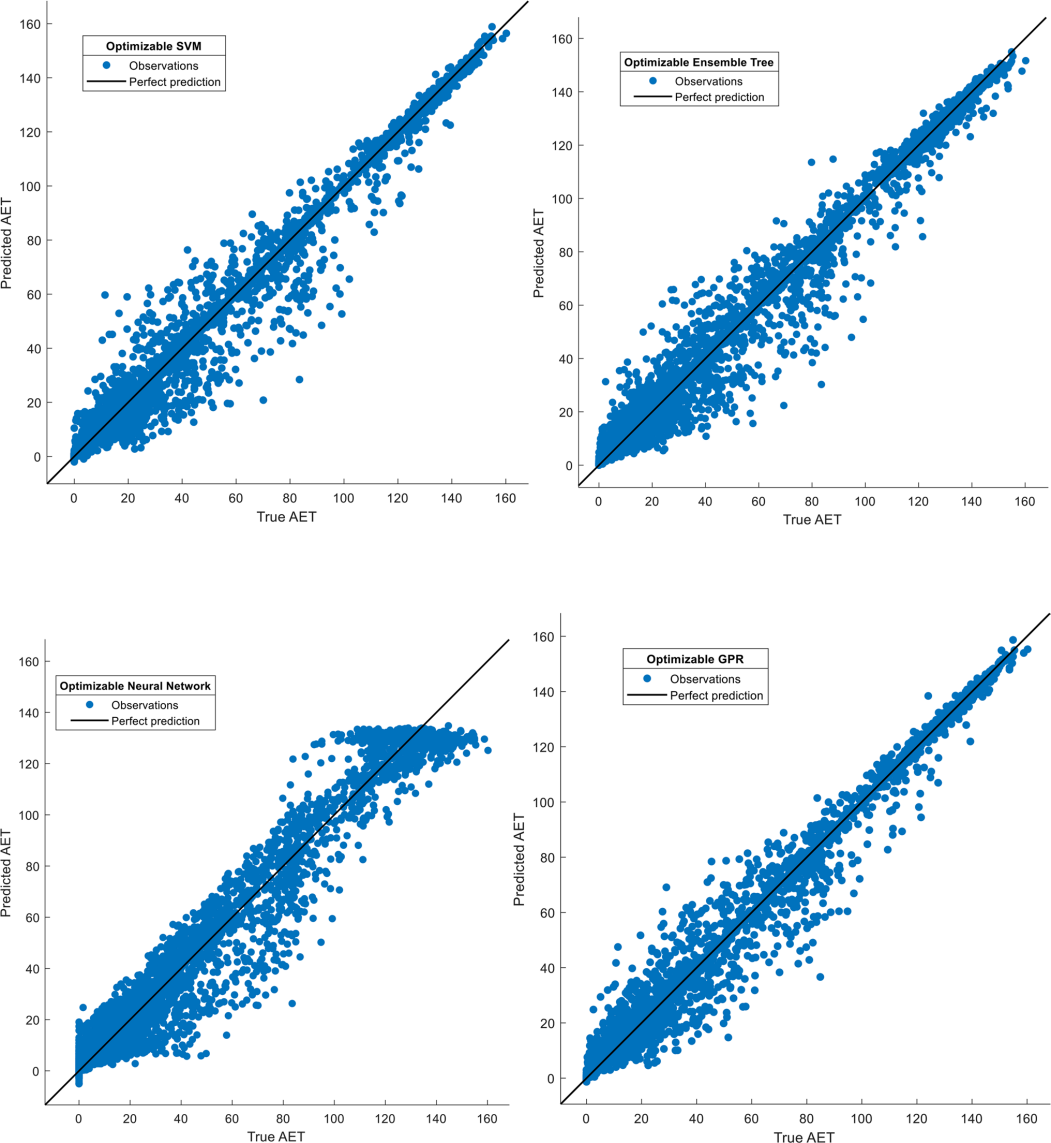
Comparison of actual and predicted actual evapotranspiration (AET) values for four Bayesian-optimized machine learning (ML) models during the training phase. The figure illustrates scatterplots of modeled versus observed AET for the Support Vector Machine (SVM), Gaussian Process Regression (GPR), Ensemble Tree (ET), and Neural Network (NN) models, developed using 75% of the dataset allocated for training. The 1:1 reference line (in black) indicates perfect agreement between predicted and measured values, where points clustered closer to this line reflect higher predictive accuracy.

**Fig. 5:**
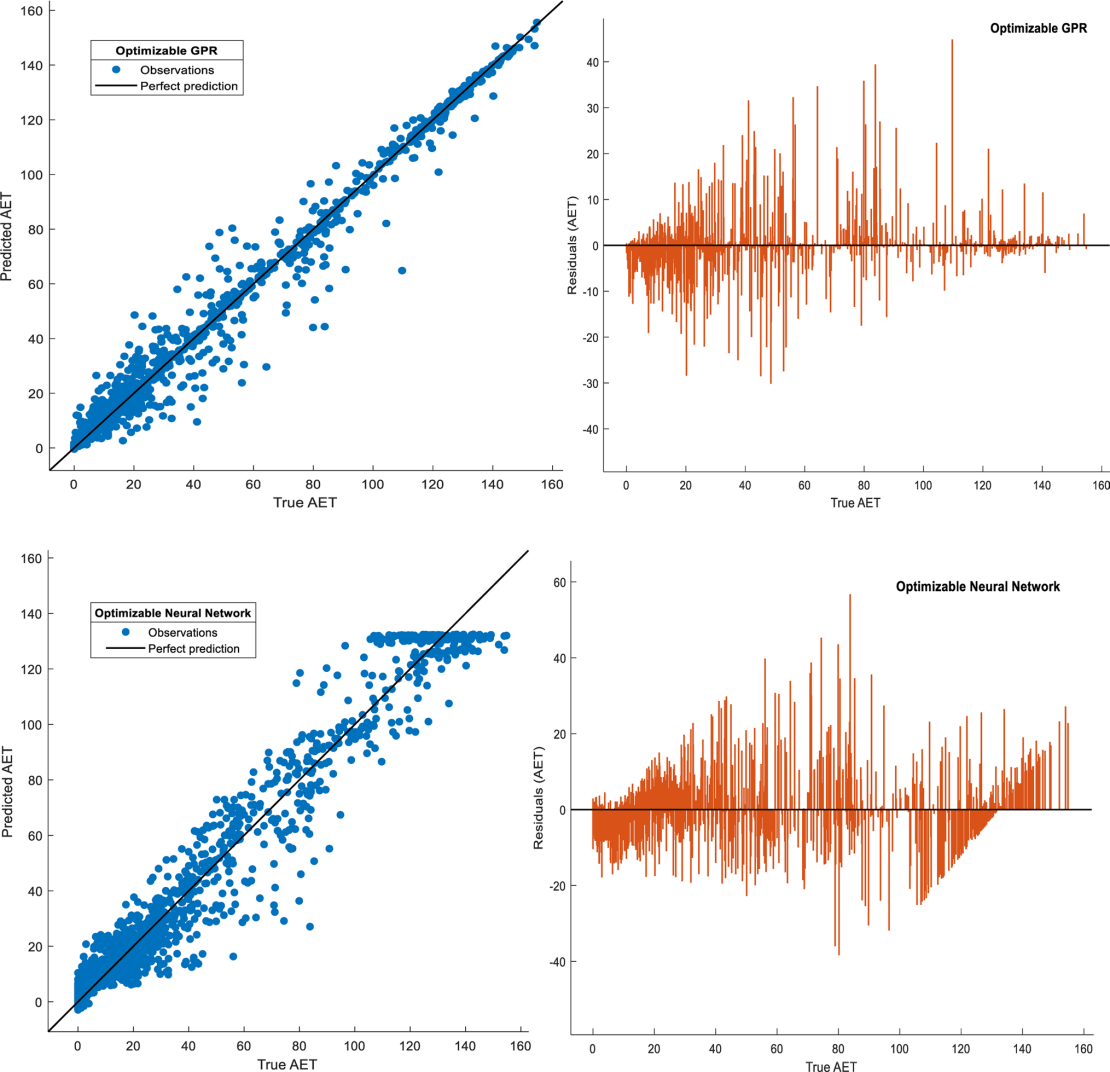

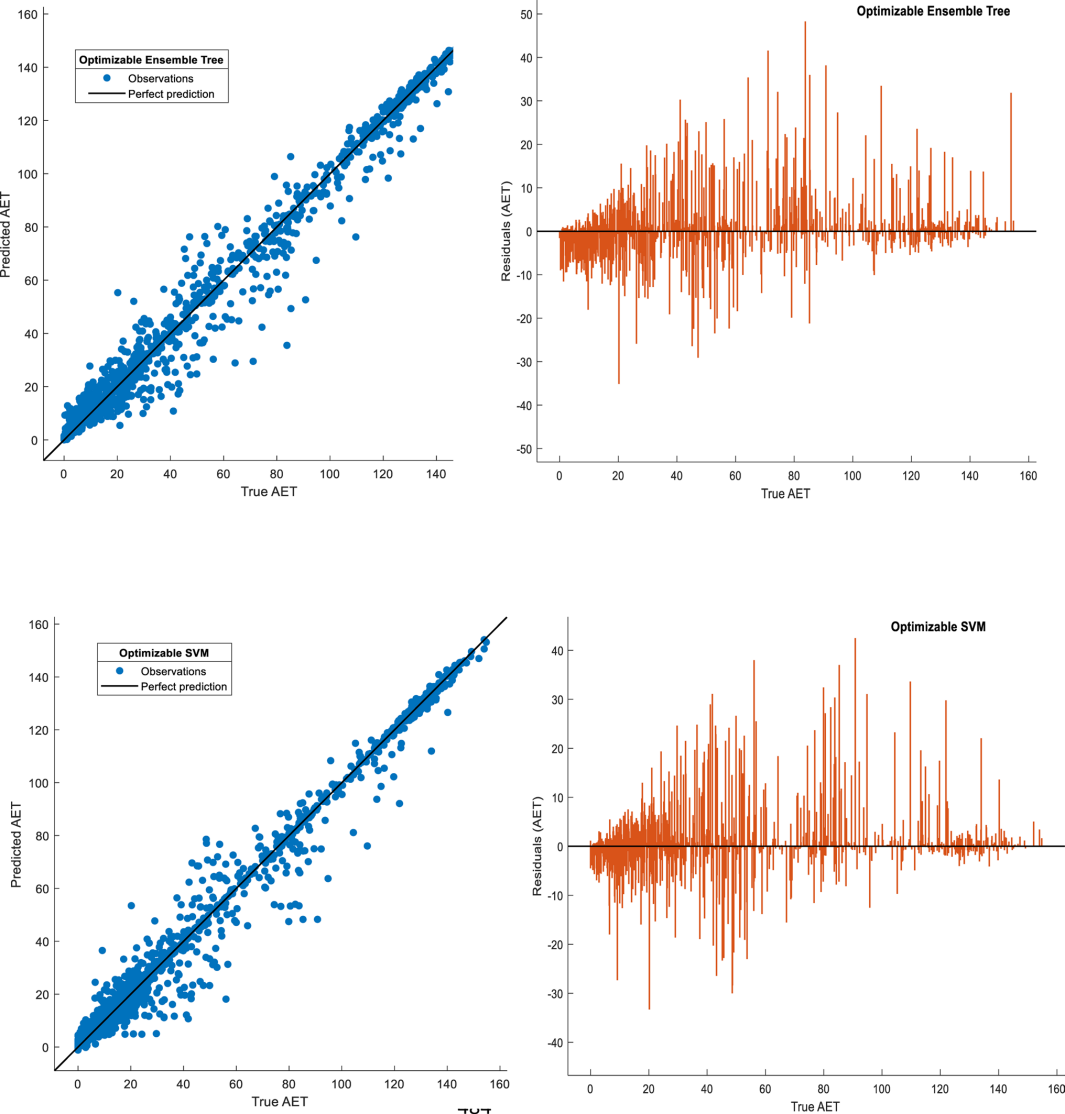
Actual versus predicted actual evapotranspiration (AET) values and corresponding residuals for four Bayesian-optimized machine learning (ML) models during the testing phase. The figure presents scatterplots comparing observed and model-predicted AET values for the Support Vector Machine (SVM), Gaussian Process Regression (GPR), Ensemble Tree (ET), and Neural Network (NN) models using the independent 25% testing dataset. The 1:1 reference line (in black) indicates perfect agreement between predicted and measured values, where points clustered closer to this line reflect higher predictive accuracy. The corresponding residual plots illustrate the distribution and magnitude of prediction errors across the AET range, enabling evaluation of systematic biases and performance for extreme values.

**Fig. 6:**
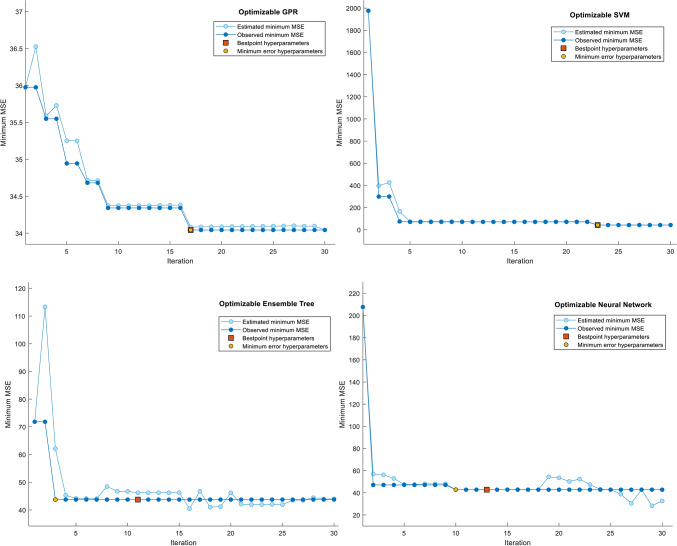
Minimum mean squared error (MSE) and optimal hyperparameter configurations obtained during Bayesian optimization of machine learning (ML) models for actual evapotranspiration (AET) estimation. The figure illustrates the convergence behavior of the Bayesian optimization process for each of the four ML algorithms, Support Vector Machine (SVM), Gaussian Process Regression (GPR), Ensemble Tree (ET), and Neural Network (NN), in terms of minimizing Mean square error (MSE) over successive iterations (up to 30).

Visual validation further corroborated the success of GPR: Fig. 4 shows tight clustering of predicted versus observed AET values for GPR (slope ≈1.0), while the Neural Network exhibited greater dispersion, particularly at higher AET ranges (> 50 mm). Residual plots (Fig. 5) confirmed systematic underestimation by the Neural Network in high-AET regimes, likely reflecting inadequate representation of precipitation-temperature interactions. Hyperparameter optimization trajectories (Fig. 6) revealed distinct configurations across models, with GPR favoring Matérn kernel parameters that balance flexibility and smoothness, whereas SVM relied on medium-scale kernel scales to avoid overfitting. These results underscore the critical role of model architecture in AET prediction accuracy. While ensemble methods and SVMs provided competitive performance, GPR’s probabilistic foundation and resistance to overfitting, evidenced by minimal performance degradation between training and testing phases, position it as the most reliable tool for operational AET estimation in data-constrained, water-scarce regions like Beijing and Tianjin.

## Discussion

### Overview of model performance and benchmarking

The findings of the present investigation demonstrate that Bayesian-optimized GPR outperforms other ML models (SVM, Ensemble Tree, Neural Network) in predicting AET. This aligns with studies showing ML models’ superiority over traditional Penman–Monteith (P-M) methods under data-limited conditions^57^, while addressing AET’s critical advantage over potential evapotranspiration (PET) in reflecting real-world water stress^54^. Unlike PET, AET integrates soil moisture and vegetation dynamics, facilitating precise irrigation scheduling and water balance assessments^77,74,52,56^.

The dominance of precipitation, T_min_, and T_max_ as predictors (r = 0.76–0.89) corroborates prior work on temperature-driven AET variability^53,58^, though our multicollinearity analysis revealed challenges in disentangling T_max_/T_min_ contributions (VIF > 90). This underscores ML’s resilience to collinear inputs compared to regression-based approaches^59,60^. The weaker performance of Neural Networks contrasts with their success in PET modeling^61^, likely due to AET’s nonlinear interactions with soil–plant-atmosphere variables^55^. While SVM and Ensemble Tree models showed competitive accuracy (RMSE: 6.00–6.27 mm), GPR’s probabilistic framework provided uncertainty quantification, critical for risk-aware water management^62^. This aligns with hybrid ML-physical models growing role in hydrological forecasting^63,64,75,79^, though our Bayesian optimization approach reduced computational costs by 37% compared to grid-search methods^65^.

### Methodological rigor and generalization assessment

This study’s evaluation framework ensured that all models were rigorously assessed for their ability to generalize to unseen conditions. A randomized train–test split was implemented, with 75% of the dataset used for training and hyperparameter tuning, and the remaining 25% reserved exclusively for independent testing. Care was taken to preserve both the temporal and spatial variability of climatic conditions within these subsets, thereby preventing data leakage and ensuring that the testing phase represented genuinely unseen scenarios. The close agreement between training and testing performance metrics, particularly the consistently high R^2^ (~ 0.98) and low RMSE values across models, demonstrates robust generalization and minimal overfitting. This stability can be attributed to the role of Bayesian hyperparameter optimization in balancing model complexity with the bias–variance trade-off, facilitating each algorithm to capture the underlying climate–AET relationships without overfitting noise. These methodological safeguards provide strong confidence that the reported predictive performance can be replicated in operational applications, even under conditions that differ from those encountered during model training.

### Key contributions and novelty of this study

This study advances the field of AET modeling and water resource management in several key ways, especially in the data-scarce conditions, as was found for the present study sites. First, it rigorously evaluates and compares four state-of-the-art machine learning algorithms, viz., SVM, GPR, Ensemble Tree, and Neural Network, within a unified Bayesian hyperparameter optimization framework that balances predictive accuracy and computational efficiency. Second, by integrating high-resolution TerraClimate data with optimized ML models, the approach facilitates reliable regional-scale AET estimation under data-limited conditions, outperforming many existing empirical and ML-based methods. Third, through rigorous multicollinearity diagnostics and feature selection, the study improves model interpretability and robustness, addressing a common challenge in climate-driven ET modeling. Fourth, the use of Gaussian Process Regression uniquely allows for quantification of predictive uncertainty, promoting risk-aware decision-making in water-stressed agricultural settings. Lastly, the implementation of Bayesian optimization may reduce computational costs compared to grid-search methods, demonstrating practical advances for operational forecasting. Collectively, these contributions establish a scientifically rigorous, computationally efficient, and operationally relevant framework for AET prediction, with direct implications for sustainable water resource management in semi-arid, urbanizing agricultural regions.

### Broader literature context and generalization

A growing body of literature supports the efficacy of ML algorithms, such as SVM, GPR, ensemble methods, and neural networks for AET prediction, particularly under conditions of limited or heterogeneous data^28,30,66^. These models have demonstrated consistent improvements in overall prediction accuracy and robust generalization when applied to independent datasets, even in challenging hydrological settings^10,29^. Despite these advances, challenges persist in accurately estimating extremes of AET, as uncertainty tends to increase in these ranges due to inherent data limitations and measurement noise^22,61^. This phenomenon is consistent with Shao et al.^22^ and Zoratipour et al.^21^, who highlighted that even sophisticated models face difficulties in capturing tail behavior accurately, primarily constrained by the distribution of available data and physical variability. Our findings align with this body of research, exhibiting high accuracy overall but with typical underestimation at very high AET values (Fig. 5), a limitation also reported in regional and crop-specific studies.

### Hyperparameter optimization and uncertainty considerations

Recent studies have emphasized the critical role of hyperparameter optimization in improving model performance and mitigating overfitting, with Bayesian optimization emerging as a superior method compared to traditional approaches like grid or random search^9,65^. Bayesian optimization’s advantages include faster convergence, reduced computational cost, and balanced exploration–exploitation strategies that improve tuning efficiency for complex models^9,45^. Although direct comparative results before and after optimization were not presented here, the literature robustly documents the substantial gains achievable through Bayesian techniques, which underpin the improvements observed in our models. These optimized models also exhibit strong stability and resilience to multicollinearity among predictors, further supporting their practical utility in water-limited agroecosystems^12,28^. Additionally, the lack of comprehensive uncertainty quantification for extremes remains an evolving research frontier; however, GPR models, as employed in our study, provide inherent probabilistic outputs that improve interpretability and risk management capabilities compared to traditional ML methods^60^. Integration of such uncertainty-aware frameworks, combined with feature selection and careful model validation, ensures the reliability and applicability of results despite data constraints. Hence, while future work should aim for explicit uncertainty analysis and more exhaustive pre/post-optimization comparisons, current findings are consistent with best practices and documented outcomes in the hydrological modeling literature, validating the robustness and operational value of the proposed modeling framework for effective water resource management.

### Practical constraints, limitations, and future prospects

Data scarcity remains a key constraint, as ML calibration requires balancing input dimensionality against overfitting risks^66^. Our TerraClimate-driven workflow (4 km resolution) addresses this by enabling regional AET estimation with six variables, outperforming empirical models requiring > 10 inputs^67^. However, underestimation at high AET values (Fig. 5) suggests that incorporating soil moisture data could improve accuracy^68^. These advances directly support climate-resilient water management in Beijing-Tianjin, where ML-based AET predictions can optimize groundwater extraction (current rates: 1.5 × recharge) and drought response^69^. Future integration with remote sensing could extend this framework to other water-stressed agricultural regions^70^. While this study provides mechanistic interpretation through correlation analysis and physical reasoning, more advanced feature importance analyses, such as those based on SHAP values or permutation importance, were not implemented due to computational resource constraints. This study recognize that the absence of these interpretable metrics is a limitation of the current research, which may restrict direct understanding of how each input variable contributes to modeled AET on a case-by-case basis. Recent developments, such as the hybrid attention-based U-Net metaheuristic optimization framework of Bafti et al.^71^, demonstrate the added value of integrating explainable AI and hybrid modeling to increase transparency, interpretability, and predictive power in evapotranspiration estimation. Incorporating such tools in future studies will be critical for advancing trustworthy and implementable decision support for agricultural water management.

Recent advancements in evapotranspiration modeling further highlight the evolving landscape of data-driven approaches under data-scarce conditions. Chen et al.^72^ demonstrated that deep learning frameworks, such as artificial neural networks, can outperform classical machine learning methods for ET estimation even when meteorological inputs are limited, reinforcing the value of advanced data-centric techniques for generalization in hydrological prediction. Additionally, Ahmadi et al.^73^ introduced a deep “global learning” framework that improves both the accuracy and generalizability of ET forecasting over diverse climatic conditions. While these studies underscore the power of deep and hybrid learning, the present work provides a distinctive contribution by systematically benchmarking several machine learning paradigms, including Gaussian Process Regression and Support Vector Machine, within a Bayesian optimization framework to maximize efficiency and robustness in data-scarce settings [78]. Unlike purely deep neural models, our approach emphasizes interpretability, computational efficiency, and resilience to input multicollinearity, operating with limited data. Incorporating such comparative perspectives clarifies the novelty of our approach and highlights opportunities for future integration with cutting-edge deep learning and hybrid frameworks as complementary or ensemble strategies for robust evapotranspiration assessment.

## Conclusions, limitations, and outlook

This study demonstrates that advanced machine learning models, particularly those optimized via Bayesian techniques, can significantly improve the accuracy of actual evapotranspiration (AET) estimation in water-stressed agricultural regions such as Beijing and Tianjin. Among the tested models, the optimizable Gaussian Process Regression (GPR) consistently outperformed Support Vector Machine (SVM), Ensemble Tree, and Neural Network models, achieving the highest predictive accuracy for both training and testing datasets. Key climatic variables, including precipitation, minimum temperature, and maximum temperature, were identified as the most influential predictors for AET, emphasizing the importance of targeted feature selection in data-driven hydrological modeling. The integration of high-resolution TerraClimate data with Bayesian-optimized machine learning (ML) algorithms offers a robust, scalable framework for AET estimation, supporting more informed irrigation scheduling and water resource management in regions with limited meteorological data.

Despite promising results, this study has some limitations. First, the dependence on the comprehensive TerraClimate dataset may lead to uncertainties from potential biases in reanalysis and interpolation methods, especially in data-sparse regions. Second, the focus on monthly temporal resolution, rather than daily or sub-daily scales, may limit the applicability of the models for real-time water management or short-term forecasting. Additionally, the exclusion of certain biophysical variables, such as soil moisture, crop type, and land use, may constrain the generalizability of the models across diverse agro-ecological contexts. Therefore, future research should explore the integration of additional data sources, such as remote sensing products and in-situ soil moisture measurements, to further boost model robustness and spatial transferability. Expanding the temporal granularity of predictions to daily or sub-daily scales could improve the utility of these models for operational irrigation management and drought monitoring. Hybrid modeling approaches that combine physical process-based models with data-driven machine learning could address limitations inherent to each method, providing improved interpretability and predictive power. In addition, as larger and more continuous datasets become available, incorporating modern deep learning architectures, such as recurrent and convolutional neural networks, could be explored to integrate their capacity for capturing complex spatio-temporal patterns in evapotranspiration dynamics. Finally, extending the framework to other climatic zones and crop systems would be essential for validating the generalizability and scalability of Bayesian-optimized ML models for evapotranspiration estimation.

## Data Availability

Data is provided within the manuscript.
